# Monitoring Hematologic Parameters in Pediatric Oncology Patients Diagnosed With Sepsis or Febrile Neutropenia: A Tertiary Center Experience

**DOI:** 10.7759/cureus.86413

**Published:** 2025-06-20

**Authors:** Florin-Mihai Radulescu, Marina Ionela Nedea (Ilie), Irina-Magdalena Dumitru

**Affiliations:** 1 Medicine, Doctoral School of Medicine, Ovidius University of Constanta, Constanta, ROU; 2 Pediatrician, "Marie Sklodowska Curie” Emergency Clinical Hospital for Children, Bucharest, ROU; 3 Department of General and Pharmaceutical Microbiology, Faculty of Pharmacy, University of Medicine and Pharmacy “Carol Davila”, Bucharest, ROU; 4 Infectious Diseases, Clinical Infectious Diseases Hospital Constanta, Constanta, ROU; 5 Medicine, Academy of Romanian Scientists, Bucharest, ROU

**Keywords:** febrile neutropenia, hemato-immunological biomarkers, hemato-oncology, pediatric oncology treatment, peditric sepsis

## Abstract

This study analyzes hematologic and inflammatory parameters in pediatric oncology patients with febrile neutropenia (FN) or sepsis, aiming to identify simple diagnostic markers. Conducted in a tertiary center, 150 infectious episodes were evaluated. Results showed statistically significant differences in leukocyte (G1: 4.95-7.03, p=0.01/G2: 4.91-7.01, p=0.01) and neutrophil counts (G1: 3.10-4.05, p=003/G2: 3.22-5.70, p=0.02) after treatment, especially in sepsis cases, with a statistically significant decrease in C-reactive protein (p=0.01) and procalcitonin (p=0.01) post-therapy. Although fibrinogen showed limited diagnostic value, the observed variation may still reflect a relevant pattern, as suggested in prior research. These findings highlight the importance of accessible biomarkers in guiding early diagnosis and management in resource-limited healthcare settings.

## Introduction

Significant advancements in oncologic therapy, including improvements in chemotherapy, radiotherapy, immunotherapy, and targeted therapies, have led to personalized treatment strategies tailored to the genetic and biological profiles of patients [1-3]. These innovations have not only enhanced treatment efficacy but also improved patient survival rates and overall quality of life. Currently, more than 80% of pediatric oncology patients achieve remission due to optimized therapeutic protocols and multidisciplinary care approaches [4-6].

Despite these advancements, oncologic treatments are often accompanied by severe adverse effects, most notably immunosuppression, which increases vulnerability to life-threatening infections. One of the most serious complications is FN, defined as the presence of fever (≥38.3°C once or ≥38.0°C sustained over one hour) in a patient with an absolute neutrophil count (NEU) below 500/mm³ or expected to fall below this threshold within 48 hours [7]. Importantly, patients with higher NEU values but impaired neutrophil function due to hematologic malignancies, chemotherapy, or recent Granulocyte Colony-Stimulating Factor (G-CSF therapy) are also included under the clinical definition of FN, given their similarly high infectious risk [8-11]. International guidelines (Infectious Diseases Society of America [IDSA], European Society for Medical Oncology [ESMO], Pediatric Oncology Group of Ontario [POGO]) recommend unified clinical management for both classical and functional forms. In pediatric oncology, a major challenge is differentiating febrile neutropenia from sepsis-a distinct, life-threatening syndrome characterized by systemic inflammation and potential multi-organ dysfunction [7-9]. Accurate and timely distinction between the two conditions is essential, as delays or misclassification can result in increased morbidity and mortality.

This study aims to analyze hematologic parameters at infection onset and recovery in pediatric oncology patients diagnosed with FN or sepsis, with the goal of identifying potential diagnostic markers and therapeutic strategies, particularly in healthcare settings with limited access to advanced diagnostic tools.

## Materials and methods

Study design and setting

This retrospective study was conducted between 2018 and 2022 within the Hematology-Oncology Department of the Emergency Clinical Hospital for Children "Maria Sklodowska Curie" in Bucharest. A total of 150 cases of sepsis and febrile neutropenia were evaluated among 94 pediatric patients diagnosed with oncologic and hematologic disorders. Data were collected from the hospital’s electronic database following approval by the Ethics Committee (Approval No. 34272/25.07.2024), ensuring compliance with ethical standards.

Patient selection and grouping criteria

This retrospective study included pediatric patients with oncologic or hematologic conditions who presented with febrile episodes between 2018 and 2022. Cases were selected from the electronic database of the Hematology-Oncology Department of the Emergency Clinical Hospital for Children "Maria Sklodowska Curie" in Bucharest, following Ethics Committee approval (Approval No. 34272/25.07.2024).

Patients were assigned to one of two distinct groups based on clinical presentation and blood culture results obtained at the time of fever onset. Group G1 included patients with fever and negative blood cultures, classified as febrile neutropenia (n = 100), while Group G2 comprised patients with fever and positive blood cultures, confirming a documented bacterial infection and thus meeting the criteria for sepsis (n = 50). It is important to note that these groups were defined prospectively at the moment of study entry, rather than retrospectively based on the patients’ clinical evolution or laboratory dynamics.

Eligible cases met at least one of the following clinical criteria: an absolute neutrophil count (ANC) below 500/mm³, as per international guidelines for severe neutropenia; or an ANC above 500/mm³ in the presence of underlying oncologic or hematologic conditions known to impair neutrophil function. The term "functional neutropenia" was used to define this latter subgroup and applied to patients who (1) had received recent myelosuppressive chemotherapy (within seven days prior to fever onset); (2) exhibited clinical signs of impaired immunity (e.g., severe mucositis, persistent unexplained fever, or rapid clinical deterioration); or (3) had recently received G-CSF therapy, which may transiently elevate ANC values while functional immune recovery remains incomplete. These criteria reflect our institutional strategy for identifying high-risk pediatric oncology patients who may benefit from early infectious workup and empirical antibiotic treatment, even in the absence of classical neutropenia. Their inclusion strengthens the real-world applicability of the febrile neutropenia concept and is supported by recent literature [12-14].

Patients were excluded if they presented with a viral etiology of fever, if blood cultures were collected solely as part of the diagnostic workup for suspected hematologic malignancy in the absence of infection (e.g., tumor fever), or if an infectious focus was identified outside the hematologic context (e.g., respiratory, urinary, or skin infections), suggesting an alternative diagnosis not aligned with the objectives of the present study.

Definition of functional neutropenia and inclusion criteria

Inclusion criteria comprised patients with an absolute neutrophil count (NEU) below 500/mm³, consistent with internationally accepted definitions of severe neutropenia and the associated infectious risk [12]. Additionally, patients with an NEU above 500/mm³ were included if they had underlying hematologic or oncologic conditions known to impair neutrophil function, thereby maintaining a high infectious risk despite preserved neutrophil counts [13]. A further inclusion criterion was the prior administration of granulocyte colony-stimulating factors (G-CSF), in which cases neutrophil counts may appear elevated while functional recovery remains incomplete, a scenario frequently described in pediatric oncology settings [14].

These clinical contexts are consistently supported by guidelines and literature, which emphasize that both quantitative and functional neutropenia carry a comparable risk for serious infections. The IDSA and ESMO guidelines recommend similar clinical management for both conditions due to their overlapping pathophysiological impact [9,12-16], a position also reflected in recent retrospective studies where both were included under the concept of febrile neutropenia [17].

Data collection and variables analyzed

The variables analyzed in this study encompassed a wide range of demographic, clinical, and laboratory data. Demographic variables included age at the time of infection, gender, place of residence, and ethnicity. Clinical background was assessed through anthropometric measurements (weight, height, and body mass index), personal medical history, family history of malignancies, and oncologic diagnosis.

Hematologic parameters were evaluated both at the time of infection (hemoglobin, total leukocyte count, lymphocytes, and neutrophils) and during post-discharge follow-up. Inflammatory markers, C-reactive protein (CRP), erythrocyte sedimentation rate (ESR), fibrinogen, and procalcitonin were recorded at the onset of infection and re-evaluated seven days after the completion of antibiotic therapy.

Surgical history was noted, particularly the presence of a central venous catheter (CVC) and any other relevant procedures. Finally, the time interval between the initiation of chemotherapy and the onset of febrile episodes or culture positivity was also documented.

Statistical analysis

Data were collected and analyzed using Microsoft Excel 2021 (Redmond, USA) and IBM Corp. Released 2019. IBM SPSS Statistics for Windows, Version 26.0. Armonk, NY: IBM Corp. Normality of quantitative values was assessed using the Kolmogorov-Smirnov and Shapiro-Wilk tests. Fisher’s exact test and Pearson’s chi-squared test were used to compare qualitative dichotomous values between the two groups. For the comparison of mean values, an independent sample T-test was applied, while the Mann-Whitney U test was used for median value comparisons. Study groups were tailored based on the calculated sample size, with a power of 80% (alpha = 0.05).

## Results

In the age group distribution, the most frequently encountered category in both groups was the 1-4 years age range. A significant proportion was also represented by patients aged 9-13 years. A statistically significant difference was observed in the mean age at the time of infection between the two groups (8.23 ± 2.6 years vs. 5.25 ± 1.2 years, p = 0.001). In the first group, the largest proportion was equally represented by patients within the aforementioned age ranges (28%), whereas in the second group, the majority of patients were aged 1-4 years (56%), a difference that was statistically significant (p=0.01). The lowest proportion was represented by patients under one year in both groups (9% vs. 4%). These details are presented in Table 1.

**Table 1:** Distribution based on age Data for continuous variables are presented as mean ± standard deviation (SD); categorical variables are shown as counts (percentages). An independent samples t-test was used for comparing mean age (t (147) = 9.60); chi-square tests were used for comparing each age group category individually. A p-value of < 0.05 was considered statistically significant.

Parameter	Group 1 (n = 100)	Group 2 (n = 50)	p-value (test)
Mean age (years)	8.23 ± 2.6	5.25 ± 1.2	p = 0.001; t(147) = 9.60
Under 1 year	9 (9%)	2 (4%)	p = 0.438; χ² = 0.60
1–4 years	28 (28%)	28 (56%)	p = 0.002; χ² = 10.01
5–8 years	14 (14%)	7 (14%)	p = 1.000; χ² = 0.00
9–13 years	28 (28%)	8 (16%)	p = 0.156; χ² = 2.01
Over 14 years	21 (21%)	5 (10%)	p = 0.147; χ² = 2.10

Table 2 presents the demographic parameters between the two groups. There were no statistically significant differences regarding sex, place of residence, or ethnicity. However, a higher proportion of male patients was observed in the first group (65% vs. 56%, p=0.28). Additionally, a relatively equal distribution of places of residence can be noted in both groups.

**Table 2:** Distribution based on gender, environment and ethnicity Data are presented as counts (percentages). Chi-square tests were applied both at the grouped level and for individual categories to assess demographic differences between Group 1 and Group 2. Global and individual p-values (a p-value of <0.05 is considered statistically significant), along with χ² statistics, are reported. A p-value of < 0.05 was considered statistically significant.

Parameter	Group 1 (n = 100)	Group 2 (n = 50)	Statistical Test Result
Gender			Global: p = 0.28; χ² = 0.80
Male	65 (65%)	28 (56%)	p = 0.372; χ² = 0.80
Female	35 (35%)	22 (44%)	p = 0.372; χ² = 0.80
Environment			Global: p = 0.56; χ² = 0.16
Rural	51 (51%)	23 (46%)	p = 0.686; χ² = 0.16
Urban	49 (49%)	27 (54%)	p = 0.686; χ² = 0.16
Ethnicity			Global: p = 0.90; χ² = 0.07
Romanian	87 (87%)	45 (90%)	p = 0.790; χ² = 0.07
Other	13 (13%)	5 (10%)	p = 0.790; χ² = 0.07

Considering the medical history of the patients, no statistically significant differences were recorded between the two groups, except for a history of infectious diseases (11% vs. 24%, p=0.03) and surgical history (17% vs. 32%, p=0.03). These data are presented in Table 3.

**Table 3:** Distribution based on medical history Data are presented as counts (percentages). Chi-square tests were applied for each medical history category to assess differences between the groups. A p-value of < 0.05 was considered statistically significant. None of the comparisons reached statistical significance.

Medical History	Group 1 (n = 100)	Group 2 (n = 50)	p-value (χ²)
Cardiac	2 (2%)	0 (0%)	p = 0.801; χ² = 0.06
Metabolic	2 (2%)	0 (0%)	p = 0.801; χ² = 0.06
Respiratory	13 (13%)	4 (8%)	p = 0.524; χ² = 0.41
ENT	11 (11%)	3 (6%)	p = 0.487; χ² = 0.48
Infectious	11 (11%)	12 (24%)	p = 0.065; χ² = 3.4
Digestive	10 (10%)	2 (4%)	p = 0.338; χ² = 0.92
Oncological	2 (2%)	1 (2%)	p = 1.0; χ² = 0.0
Gynecological	0 (0%)	1 (2%)	p = 0.723; χ² = 0.13
Renal	2 (2%)	1 (2%)	p = 1.0; χ² = 0.0
Neurologic	4 (4%)	1 (2%)	p = 0.872; χ² = 0.03
Alergologic	2 (2%)	1 (2%)	p = 1.0; χ² = 0.0
Psychiatry	1 (1%)	1 (2%)	p = 1.0; χ² = 0.0
Surgical	17 (17%)	16 (32%)	p = 0.06; χ² = 3.54
Malignancy	9 (9%)	6 (12%)	p = 0.773; χ² = 0.08
History of radiotherapy	2 (2%)	3 (6%)	p = 0.421; χ² = 0.65

The hematologic parameters analyzed between the two groups, both at infection and during post-discharge follow-up, are presented in Table 4. A statistically significant difference in neutrophil values at follow-up was detected between the two groups (4.05 ± 0.82 vs. 5.6 ± 1.31, p=0.04).

**Table 4:** Analysis of hematologic parameters at admission and post-discharge follow-up HB: Hemoglobin, LEU: Leukocytes (White blood cells), LYMPH: Lymphocytes, NEU: Neutrophils Data are presented as mean ± standard deviation (SD). Welch's t-test was used to compare each hematologic parameter between the groups. t(df) values and corresponding two-tailed p-values are reported. A p-value of < 0.05 was considered statistically significant.

Parameter (unit)	Group 1 (n = 100)	Group 2 (n = 50)	t(df)	p-value
HB at infection (g/dL)	9.49 ± 1.60	9.59 ± 1.70	t(93) = -0.35	p = 0.727
LEU at infection (×10³/μL)	5.21 ± 1.95	5.36 ± 1.41	t(129) = -0.54	p = 0.59
LYMPH at infection (×10³/μL)	1.41 ± 0.24	1.64 ± 0.95	t(52) = -1.69	p = 0.097
NEU at infection (×10³/μL)	3.18 ± 1.90	3.67 ± 1.74	t(106) = -1.58	p = 0.117
HB at control (g/dL)	9.51 ± 1.40	9.56 ± 1.32	t(103) = -0.21	p = 0.834
LEU at control (×10³/μL)	7.03 ± 1.47	7.00 ± 1.54	t(94) = 0.11	p = 0.913
LYMPH at control (×10³/μL)	1.75 ± 0.33	2.40 ± 1.12	t(53) = -4.02	p = 0.0
NEU at control (×10³/μL)	4.05 ± 0.82	5.60 ± 1.31	t(69) = -7.65	p = 0.0

While this result reaches statistical significance, the absolute difference of approximately 1.5 × 10³ /μL suggests a trend toward improved hematologic recovery in the sepsis group; however, this value alone may not represent a decisive clinical threshold. Thus, interpretation should consider both the broader clinical picture and other correlated parameters.

The therapeutic response was evaluated based on hematologic parameters by monitoring their dynamics from infection to follow-up, individually for each group. Statistically significant differences were detected in leukocyte values in both groups (p=0.01) and in neutrophil values (p=0.03, p=0.02). A significant increase in lymphocytes was observed in Group 2 (1.67 (±1.00) vs. 2.34 (±1.13), p=0.04). These details are presented in Table 5. 

**Table 5:** Comparative analysis of dynamic parameters between the two groups Data are presented as mean ± standard deviation (SD). Group comparisons were performed using independent samples t-tests; t(df) values are reported in the table. A p-value of < 0.05 was considered statistically significant. G1: group 1; G2: group 2 HB: Hemoglobin, LEU: Leukocytes (White blood cells), LYMPH: Lymphocytes, NEU: Neutrophils

Parameter (unit)	Infection (mean ± SD)	Control (mean ± SD)	p-value (t(df))
HB (g/dL) -G1	9.49 ± 1.60	9.51 ± 1.40	p = 0.88; t(195) = -0.09
LEU (×10³/μL) - G1	4.95 ± 0.77	7.03 ± 1.47	p = 0.01; t(150) = -12.53
LIMF (×10³/μL) - G1	1.41 ± 0.25	1.75 ± 0.33	p = 0.20; t(184) = -8.21
NEU (×10³/μL) - G1	3.10 ± 0.82	4.05 ± 0.82	p = 0.03; t(198) = -8.19

Parameter(unit)	Infection (mean ± SD)	Control (mean ± SD)	p-value (t(df))
HB (g/dL) - G2	9.58 ± 1.72	9.55 ± 1.33	p = 0.88; t(92) = 0.10
LEU (×10³/μL) - G2	4.91 ± 0.78	7.01 ± 1.62	p = 0.01; t(71) = -8.26
LIMF (×10³/μL) - G2	1.67 ± 1.00	2.34 ± 1.13	p = 0.04; t(97) = -3.14
NEU (×10³/μL) - G2	3.22 ± 1.01	5.70 ± 2.13	p = 0.02; t(70) = -7.44

Considering the inflammatory parameters at the time of infection and follow-up, no statistically significant differences were observed in fibrinogen levels. However, a significant improvement in procalcitonin as well as CRP levels was recorded within the sample (p=0.001). These details are presented in Table 6.

**Table 6:** Comparative analysis of inflammatory markers between the two samples Data are presented as mean ± standard deviation (SD). Welch's t-test was applied to compare inflammation markers between the infection and control groups. t(df) values and corresponding two-tailed p-values are reported. All comparisons were statistically significant at p < 0.001.

Parameter (unit)	Infection (n = 100)	Control (n = 50)	t(df)	p-value
Fibrinogen (mg/dL)	288.00 ± 22.02	214.15 ± 20.82	t(103) = 20.09	p < 0.001
Procalcitonin (ng/mL)	21.20 ± 4.64	0.89 ± 0.45	t(103) = 43.37	p < 0.001
CRP levels (mg/dL)	17.40 ± 4.91	5.64 ± 0.89	t(111) = 23.2	p < 0.001

Despite the lack of statistical significance, this approximate 74 mg/dL decrease may carry clinical implications when interpreted alongside the consistent post-therapy reductions observed in procalcitonin and CRP levels.

## Discussion

Procalcitonin and CRP have emerged as valuable biomarkers in distinguishing febrile neutropenia from sepsis, given their high specificity and sensitivity in detecting bacterial infections [15,16]. Procalcitonin, in particular, is a well-established marker for systemic bacterial infections and has been shown to correlate with infection severity, aiding clinicians in differentiating sepsis from other inflammatory conditions [16]. Similarly, fibrinogen plays a role in the acute phase response, and its elevated levels may indicate a heightened inflammatory state, helping stratify infection risk [17]. However, it is essential to interpret these variations in conjunction with the patient's clinical trajectory, as modest changes in fibrinogen levels may not independently justify diagnostic or therapeutic shifts. Although the difference in fibrinogen levels between the two groups did not reach statistical significance, a noticeable variation in values was observed. These variations may reflect different baseline values and inconsistent inflammatory responses. As such, fibrinogen’s utility should be interpreted cautiously but remains potentially relevant and worth exploring further in prospective settings.

Neutropenia in pediatric oncology patients is characterized by a profound decrease in neutrophil count, as well as a reduction in neutrophil functionality, leading to an increased risk of opportunistic infections. However, the absence of overt clinical symptoms in some cases makes early detection of serious infections challenging [18]. In contrast, sepsis presents with more systemic inflammatory responses, such as hemodynamic instability and multi-organ dysfunction, necessitating prompt intervention [19]. The dynamic changes in leukocyte and neutrophil counts observed in our study underscore the importance of hematologic monitoring in both febrile neutropenia and sepsis. It is also important to highlight that, beyond statistical significance, some of the observed variations, especially in leukocyte and neutrophil counts, suggest moderate effect sizes. These findings can aid in diagnostic stratification, yet their clinical applicability on their own remains limited without corroborating clinical data. As such, our analysis aims to strike a balance between statistical interpretation and real-world clinical impact.

This study aimed to document the value ranges of key hematologic and inflammatory markers both at the onset of infection and after clinical resolution. The observed dynamics of neutrophil counts, CRP, and procalcitonin offer insight into how these parameters evolve across different infectious contexts. Although retrospective, these findings may serve as a basis for future prospective investigations into early risk stratification.

It is important to note that in the researched cohort, both classic febrile neutropenia cases and situations where neutrophil counts are within normal limits but functional impairment is present were included. This study showed that functional deficiency can significantly impact the response to the infection, as demonstrated in the cases of children with hematological malignancies [20]. Additionally, the use of treatment with filgrastim for these patients has been shown to enhance neutrophil production and improve their functional capacity, thereby reducing infection-related complications [21]. Patients with ANC above 500/mm³ but recent myelosuppressive treatment, significant clinical signs of immune compromise, or recent G-CSF use often demonstrated clinical trajectories similar to those of patients with severe neutropenia, supporting their inclusion in the febrile neutropenia group.

Our findings are in line with previous studies that have emphasized the role of leukocyte and neutrophil counts in predicting the progression of febrile neutropenia. For example, [22] demonstrated that persistently low neutrophil counts were associated with higher infection-related complications, a trend also observed in our cohort. Additionally, our study corroborates research [23], which suggested that early risk stratification using inflammatory markers could guide more effective antibiotic administration and reduce morbidity.

In resource-limited settings where advanced molecular diagnostic tools are not readily available, reliance on hematologic and inflammatory markers becomes crucial. The combined use of leukocyte count, neutrophil percentage, fibrinogen, and procalcitonin can serve as a cost-effective strategy for identifying high-risk patients [24]. Future studies should aim to develop simplified diagnostic algorithms incorporating these markers, allowing for timely and accurate differentiation between febrile neutropenia and sepsis even in healthcare facilities with limited resources. This study highlights that accessible biomarkers such as CRP and procalcitonin may support the early identification of pediatric oncology patients at risk of severe infection. Their dynamic changes are associated with clinical evolution and may assist in the timely initiation of empirical antibiotic therapy, especially in resource-limited settings. While not definitive diagnostic tools, these markers can guide clinical decisions and warrant further validation in prospective studies.

Further investigations should also explore the integration of artificial intelligence and machine learning models to improve predictive accuracy based on hematologic and inflammatory markers. With continued advancements in technology, incorporating these models into clinical decision-making could enhance early detection and management, ultimately reducing infection-related mortality in pediatric oncology patients [9].

By contextualizing our study with previous research and expanding diagnostic applications, we provide a more comprehensive framework for addressing febrile neutropenia and sepsis in pediatric oncology. Future research should focus on refining predictive models, integrating novel biomarkers, and tailoring treatment protocols to individual patient risk profiles.

One of the limitations identified in our study, also reported in the scientific literature, is the relatively low number of cases recorded during the analyzed timeframe, possibly influenced by the restrictions imposed during the COVID-19 pandemic (2020-2022) [25]. As a formal power analysis was not conducted prior to the study, the target sample size and power were estimated based on feasibility considerations, which may be considered another limitation of our study.

## Conclusions

This study highlights the significance of hematologic and inflammatory markers in differentiating febrile neutropenia from sepsis in pediatric oncology patients. Procalcitonin, along with leukocyte and neutrophil counts, demonstrates potential as a cost-effective and accessible biomarker, particularly in healthcare centers with limited diagnostic resources. Our findings align with existing literature, reinforcing the importance of early risk stratification to optimize therapeutic strategies and reduce infection-related complications. Despite not having a statistically significant difference, a tendency towards increased values of fibrinogen can be highlighted, prompting further research in that regard.

Further research should focus on refining diagnostic algorithms that integrate these biomarkers, allowing for improved clinical decision-making. The application of machine learning models and predictive analytics may enhance early detection and treatment personalization. By improving the accuracy of early diagnosis and treatment initiation, the morbidity and mortality associated with infections in immunocompromised pediatric oncology patients can be significantly reduced.
